# Potential Targets and Mechanisms of Bitter Almond-Licorice for COVID-19 Treatment Based on Network Pharmacology and Molecular Docking

**DOI:** 10.2174/0113816128265009231102063840

**Published:** 2023-12-13

**Authors:** Qiwei Hong, Xinyue Shang, Yanan Wu, Zhenlin Nie, Bangshun He

**Affiliations:** 1 Department of Laboratory Medicine, Nanjing First Hospital, China Pharmaceutical University, Nanjing, China;; 2 Department of Clinical Pharmacy, School of Basic Medicine and Clinical Pharmacy, China Pharmaceutical University, Nanjing, China;; 3 Department of Laboratory Medicine, Nanjing First Hospital, Nanjing Medical University, Nanjing, China

**Keywords:** Bitter almond, licorice, COVID-19, network pharmacology, molecular docking, traditional chinese medicine

## Abstract

**Background:**

The outbreak of Corona Virus Disease 2019 (COVID-19) has resulted in millions of infections and raised global attention. Bitter almonds and licorice are both Traditional Chinese Medicines (TCM), often used in combination to treat lung diseases. Several prescriptions in the guidelines for the diagnosis and treatment of coronavirus disease 2019 (trial version ninth) contained bitter almond-licorice, which was effective in the treatment of COVID-19. However, the active ingredients, drug targets and therapeutic mechanisms of bitter almonds-licorice for the treatment of COVID-19 remain to be elucidated.

**Methods:**

The active ingredients and targets were derived from the Traditional Chinese Medicine Systems Pharmacology (TCMSP). Meanwhile, targets associated with COVID-19 were obtained from the GeneCards database, PharmGkb database and DrugBank database. Then, the potential targets of bitter almond-licorice against COVID-19 were screened out. Protein-protein interaction (PPI) networks and core targets were analyzed through the String database and Cytoscape software. In addition, gene ontology (GO) and Kyoto Encyclopedia of Genes and Genomes (KEGG) pathway enrichment analyses were performed based on potential targets using R statistical software. Finally, molecular docking was used to validate the binding of the active ingredients to the core targets.

**Results:**

The results of the TCMSP database showed that the bitter almond-licorice had 89 active components against COVID-19, involving 102 targets. PPI network and core target analysis indicated that IL-6, TNF, MAPK1, and IL1B were the key targets against COVID-19. In addition, GO and KEGG enrichment analysis showed that the bitter almond-licorice were involved in various biological processes through inflammation-related pathways such as TNF signaling pathway and IL-17 signaling pathway. Finally, molecular docking approaches confirmed the affinity between the active components of the bitter almond-licorice and the therapeutic targets.

**Conclusion:**

The bitter almond-licorice could be used to treat COVID-19 by inhibiting inflammatory responses and regulating cellular stress. This work is based on data mining and molecular docking, and the findings need to be interpreted with caution.

## 1 INTRODUCTION

In December 2019, Corona Virus Disease 2019 (COVID-19) was identified in China, which was caused by a new virus named SARS-CoV-2, an enveloped RNA virus which posed a serious threat to humans and became a public health event [1, 2]. Globally, more than 593 million cases and 6.4 million deaths have been reported by 21 August 2022 [3]. Historically, Traditional Chinese Medicine (TCM) has always been used in the prevention and treatment of plagues. China has formulated nine editions of the guidelines for the diagnosis and treatment of coronavirus disease 2019 [4], in which the treatment methods of Chinese medicine have been gradually improved, including treatment, typing and classification, recommended prescriptions and medicines, which have played an important role in the prevention and treatment of COVID-19.

The couplet medicine is the joint application of two herbal medicines, and its law and mechanism are one of the foundations of modern Chinese medicine compound research [5]. Studies on the medication rules of TCM in the treatment of COVID-19 [6, 7] have reported that bitter almond (a herb) is the most frequently used medicine. In the medical practice of TCM, bitter almond as the main medicine, often combined with other medicines, among which licorice (a herb) is the most commonly used medicine [8]. In the guidelines for the diagnosis and treatment of coronavirus disease 2019 (trial version ninth) [9], the administration of Lianhua Qingwen granules (commercial Chinese medicine) and XuanFeiBaiDu Decoction (Chinese medicine consisting of herbs prescribed by the doctor of TCM) containing bitter almond and licorice were also recommended to treat COVID-19. Based on TCM theory, Licorice is sweet and used to clear heat, a specific symptom term which frequently caused by inflammatory response or viral invasion, often applied in the treatment of cough and phlegm [10], while bitter almond is bitter and toxic and used in relieving cough, asthma [8]. A study [11] has reported that licorice can reduce the toxicity of bitter almond and reduce the content of amygdalin.

Since the “multi-component, multi-pathway, multi-target, and holistic regulation” of TCM in the human body fits well with the systematic analysis of “drug, target, pathway, and disease” in network pharmacology, it is practical to explore the molecular mechanisms of bitter almond-licorice for the treatment of COVID-19 using the network pharmacology approach. Bitter almond-licorice is frequently used in the treatment of COVID-19 [12]. The administration of LianHuaQingWen capsule containing bitter almond-licorice, has achieved a satisfactory outcome in the treatment of COVID-19 [13]. However, the mechanisms underlying bitter almond-licorice in the clinical application are still unclear. This study therefore aimed at ascertaining the possible mechanisms of the bitter almond-licorice against COVID-19. We screened the main active ingredients, predicted the targets of the active ingredients, and analyzed the key targets and pathways based on network pharmacology. The study design and workflow are presented in Fig. (**1**).

## 2 MATERIALS AND METHODS

### 2.1 Screening for Active Components and Target

All ingredients of bitter almond and licorice were retrieved from the natural product databases for Chinese herbal medicines: Traditional Chinese Medicine Systems Pharmacology (TCMSP) database (https://old.tcmsp-e.com/tcmsp.php) [13]. We selected the “Herb name” for bitter almond and licorice, respectively. Oral bioavailability (OB) and drug-likeness (DL) are two important pharmacokinetic parameters. We filtered active ingredients with OB ≥ 30% and DL ≥ 0.18 [14, 15] and, according to these compounds searched the related targets. However, the target names in TCMSP are not standard, so we obtain the gene symbols from the Uniprot database (http://www.uniprot.org/) [16].

### 2.2 Collection of COVID-19 Related Gene Set

Using COVID-19 as the keyword, a search in the GeneCards database (https://www.genecards.org/) [17], PharmGkb database (https://www.pharmgkb.org/) [18], DrugBank database (https://www.drugbank.ca/) [19] was conducted to obtain the relevant disease targets, and then, a merged set was drawn based on all the collected gene targets of COVID-19, which were further standardized using the Uniprot database.

### 2.3 Screening of the Potential Targets

To explore potential targets for bitter almond-licorice treatment of COVID-19, we drew a Venn diagram [20] by intersecting the bitter almond-licorice target set and the COVID-19-related gene set to reveal the co-genes between the active ingredients and COVID-19.

### 2.4 Network of Herb-ingredient-target Construction

To investigate the relationship between the components and targets of bitter almond-licorice in the treatment of COVID-19, we constructed a herb-ingredient-target network by the Cytoscape software (version 3.9.1), which is composed of nodes and edges, representing a molecule (ingredient or target) and a biological relationship between two nodes, respectively.

### 2.5 Protein-protein Interaction (PPI) and Targets Analysis

Proteins usually achieve biological functions through interactions with other proteins. To identify proteins interaction in the administration of these two paired herbs, we predicted PPI with the drug-disease common genes processed using the String database (https://string-db.org/) [21] with the protein type set to Homo sapiens, and the confidence score not less than 0.9. To better explore core targets, we used CytoNCA (version 3.9.1) to identify key targets based on Betweenness Centrality (BC), Closeness Centrality (CC), Degree Centrality (DC), Eigenvector Centrality (EC), Local Average Connectivity (LAC), and Network Centrality (NC). BC reflects the degree of cohesion of nodes in the network through the shortest path number of nodes. CC calculates the sum of distances from a node to all other nodes, reflecting the proximity of one node to other nodes. DC is the most direct indicator for analyzing the importance of nodes in a network, representing the total number of other nodes connected to a node. EC reflects the importance of other nodes connected to this node. LAC evaluates the relationship between a node and its neighbors. NC is also an index to evaluate the importance of nodes in the network. The nodes whose all six scores are above the median value could be considered a hub.

### 2.6 Gene Ontology (GO) Enrichment and Kyoto Encyclopedia of Genes and Genomes (KEGG) Pathway Analysis

To identify biological processes and molecular interactions associated with selected common genes, GO enrichment and KEGG pathway analysis were performed using R (version 4.2.1), with the top thirty items whose *p*-value < 0.05 selected.

### 2.7 Molecular Docking

To verify that the screened active ingredients can be used to treat COVID-19, we performed molecular docking of the active ingredients with angiotensin-converting enzyme 2 (ACE2), a potential therapeutic target of COVID-19, and selected hydroxychloroquine [22], a potential antiviral drug against COVID-19, as a positive control.

To further validate the effective binding of the candidate ingredients to the core targets, we used AutoDock-Vina (version 1.1.2) for molecular docking. The structure of the drug small molecule was downloaded from the Pubchem database (https://pubchem.ncbi.nlm.nih.gov/) [23], while the three-dimensional (3D) structure of the target protein was downloaded from PDB Database (https://www.rcsb.org/) [24]. Then, we used PyMol (version 4.6.0) to dehydrate and remove the ligand. The target protein was hydrogenated and converted to pdbqt format (a file format) for the docking, and the drug small molecule was saved in pdbqt format with minimal structural energy by ChemBioDraw 3D (version 14.0.0). Molecular docking was performed by AutoDock-Vina after defining the grid at the active site of the receptor protein. When a drug molecule ligand combines with a target to form a conformational stability, the structure becomes more stable as the energy lowers. Finally, the docking results were visualized by Discovery Studio (version 19.1.0).

## 3 RESULTS

### 3.1 Chemical Database Construction

A total of 104 active portions (listed in affiliated file 1, 92 ingredients from licorice, 19 from bitter almond and 7 both) were considered for further study after removing the unqualified ingredients. The active ingredient-related targets were sorted out, excluding duplicate targets, and consequently, 246 targets were screened.

### 3.2 COVID-19 Related Targets

**Fig. (2):**
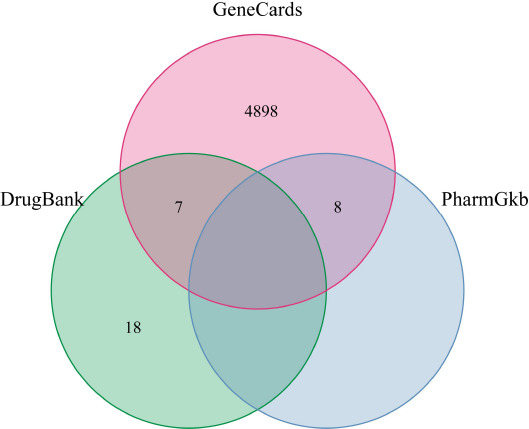
Target source of COVID-19. Identification of the COVID-19-related genes by taking a union of all the results from 3 database.

We obtained 4913 COVID-19-related disease targets from the GeneCards database, 25 from the DrugBank database, and 8 from the pharmGkb database. After removing the duplicated targets, 4931 COVID-19-related disease targets were retained for further study (Fig. **2**).

### 3.3 Screening of the Potential Targets

**Fig. (3):**
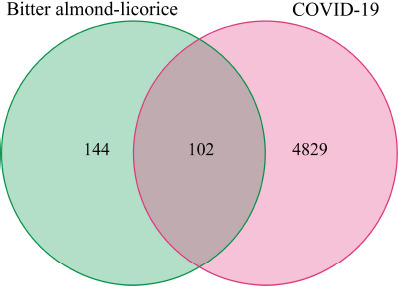
The 102 overlapping genes between COVID-19 and bitter almond-licorice.

A total of 102 intersected gene targets were obtained based on 246 active ingredient-related targets and 4829 COVID-19-related disease targets, which are possible key gene targets of the bitter almond-licorice for the treatment of COVID-19 (Fig. **3**), such as IL10 and IL6. These targets were potential targets of bitter almond-licorice against COVID-19 (Table **1**). These 102 targets corresponded with 89 active ingredients of bitter almond-licorice.

### 3.4 Herb-ingredient-target Network

**Fig. (4):**
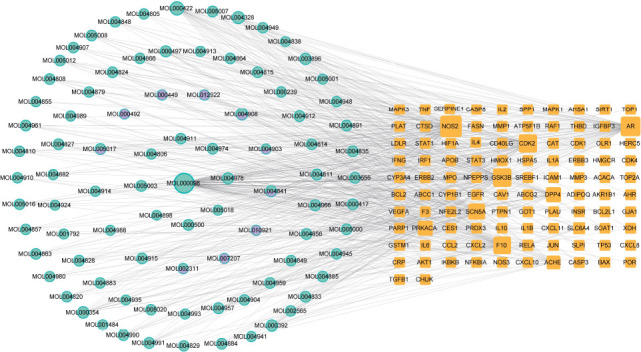
Herb-ingredient-target network of bitter almond-licorice against COVID-19. The green circles represent ingredient of licorice, the purple circles represent ingredient of bitter almond pair, the orange squares represent targets.

The component-target network consisted of 191 (102 targets and 89 compounds) nodes and 585 edges (Fig. **4**). The green circles represent ingredient of licorice, the purple circles represent the ingredient of bitter almond, and the orange squares represent targets. The results showed that the compound nodes had a median DC value of 6 and 47 compounds are higher than the median, including quercetin, kaempferol, naringenin, Glabridin, isorhamnetin, formononetin, 7-Methoxy-2-methyl isoflavone, licochalcone a, 2-[(3R)-8,8-dimethyl-3,4-dihydro-2H-pyrano [6,5-f]chromen-3- yl]-5-methoxyphenol, 7-Acetoxy-2-methylisoflavone, Glabrone, Glepidotin A, Glyasperin C, Glyasperins M, HMO, Licoagrocarpin, Vestitol, (2S)-6-(2,4-dihydroxyphenyl)-2-(2-hydroxypropan-2 -yl)-4-methoxy-2,3-dihydrofuro [3,2-g]chromen-7-one, 1-Methoxy- phaseollidin, 3'-Hydroxy-4'-O-Methylglabridin, 3'-Methoxygla- bridin, Eurycarpin A, Gancaonin A, glyasperin B, Glycyrrhiza flavonol A, Glypallichalcone, Licoagroisoflavone, licoisoflavanone, Lupiwighteone, Phaseolinisoflavan, Quercetin der., Semilicoisoflavone B, (E)-1-(2,4-dihydroxyphenyl)-3-(2, 2-dimethylchromen-6-yl)prop-2-en-1-one,3-(2,4-dihydroxyphenyl)-8-(1, 1-dime- thylprop-2-enyl)-7-hydroxy-5-methoxy-coumarin, 3-(3,4-dihydroxyphenyl)-5,7-dihydroxy-8-(3-methylbut-2-enyl)chromone, 5,7-dihydroxy-3-(4-methoxyphenyl)-8-(3-methylbut-2-enyl)chromone, 7, 2',4'-trihydroxy-5-methoxy-3-arylcoumarin, Calycosin, Gancaonin G, Glabrene, glyasperin F, Glyzaglabrin, kanzonols W, Licoisoflavone B, Medicarpin, Odoratin and shinpterocarpin which suggested that these compounds paly a relatively important role in bitter almond-licorice to exert anti-COVID-19. Among those, quercetin, naringenin and kaempferol acted on multiple targets, which may be the key active ingredients (Table **2**).

### 3.5 Ingredient-COVID-19 PPI Network

**Fig. (5):**
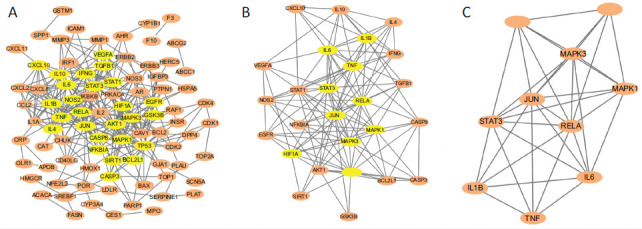
Protein-protein interaction (PPI) networks. The orange ellipses represent the target, the yellow ellipses represent the selected core target. (**A**) Original PPI network consists of 88 nodes and 331 edges. (**B**) Subnetwork after first selection consists of 26 nodes and 126 edges. (**C**) Final network after second selection consists of 10 nodes and 34 edges.

The identified 102 component-COVID-19 co-genes were imported into the String database to obtain a PPI network with a confidence level > 0.9 (Fig. **5A**), consisting of 88 nodes and 331 edges. After being analyzed by CytoNCA, we filtered the core targets based on the scores of BC, CC, DC, EC, LAC, and NC. The target nodes have a median BC of 41.5262, a median CC of 0.1526, a median DC value of 5, a median EC value of 0.0427, a median LAC of 2, and a median NC of 2.6667. 26 genes with high correlation were selected to construct a subnetwork with 26 nodes and 126 edges (Fig. **5B**). To focus the key targets of the 26 genes to further screen the core targets, we ranked the subnetwork and re-filtered these 26 genes to obtain 10 key targets whose scores were higher than a median BC of 8.8276, a median CC of 0.5814, a median DC value of 8, a median EC value of 0.1636, a median LAC of 4.7186, and a median NC of 5.9946, including IL-6, MAPK1, TNF, IL1B, HIF1A, TP53, RELA, MAPK3, STAT3, and JUN, which may play more critical roles in the target network, and finally, a final PPI network was constructed with 10 nodes and 34 edges (Fig. **5C**).

### 3.6 GO Functional Enrichment and KEGG Pathway Enrichment Analysis

**Fig. (6):**
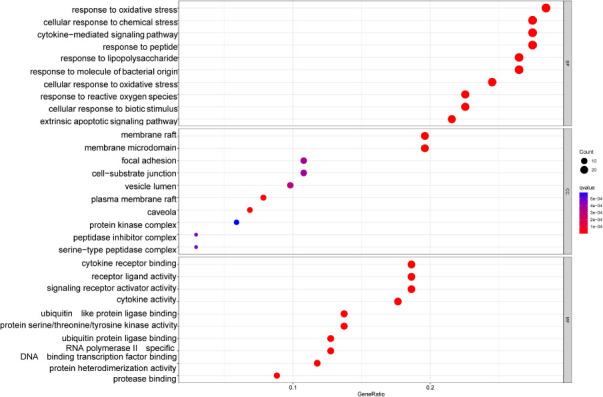
GO analysis of the 102 potential targets of bitter almond-licorice associated with COVID-19. The horizontal axis (Gene Radio) represents the ratio of the targets in each term to the total number of targets; the size of the bubble represents the number of targets in each term; and the color from red to blue suggests the p-value from small to large.

GO functional enrichment consists of three parts: biological process (BP), cellular component (CC), and molecular function (MF). We displayed the top 10 terms that were most relevant respectively (Fig. **6**). In the BP part, the active compounds of bitter almond-licorice are mainly through cellular response to chemical stress, cellar response to oxidative stress, response to reactive oxygen species, cellular response to biotic stimulus, and so forth. These processes involve changes in the state or activity of cells or organisms caused by stimulation, which is consistent with the stress that occurs in the body after virus infection. The CC section revealed that the treatment of bitter almond-licorice for COVID-19 was significantly related to membrane raft and membrane microdomain, suggesting that bitter almond-licorice plays an anti-COVID-10 role mainly by acting on the cell membrane. The result of MF shown that anti-COVID-19 function of bitter almond-licorice was associated with cytokine receptor binding, receptor ligand activity, signaling receptor activator activity, and cytokine activity, indicating that bitter almond-licorice affected cytokine activity and receptor ligand binding to influence the physiological and biochemical processes of the body.

**Fig. (7):**
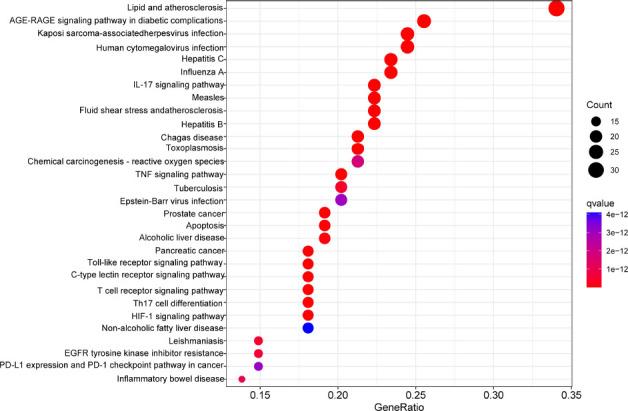
The KEGG pathway enrichment analysis for the 102 potential targets. The horizontal axis (Gene Radio) of the bubble diagram represents the ratio of the targets involved in each pathway to the total number of targets; the size of the bubble represents the number of targets in each pathway; and the color from red to blue suggests the P-value was from small to large.

As for KEGG pathway enrichment analysis, the top 30 pathways were shown with P-value from smallest to largest (Fig. **7**). The result revealed that 102 potential genes were highly associated with multiple immune response and inflammation-related pathways, including IL-17 signaling pathway, TNF signaling pathway, and Th17 cell differentiation. In addition, those targets are also related to AGE-RAGE signaling pathway and other virus infection.

### 3.7 Molecular Docking

**Fig. (8):**
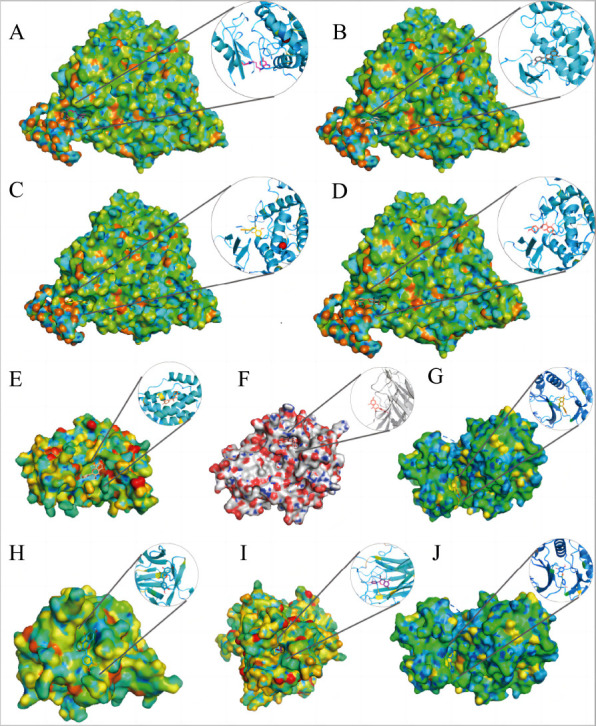
The docking complex of the components and targets. (**A**) hydroxychloroquine-ACE2 with binding energy -6.4 kcal/mol, (**B**) quercetin-ACE2 with binding energy -6.3 kcal/mol, (**C**) kaempferol-ACE2 with binding energy -6.2 kcal/mol, (**D**) naringenin-ACE2 with binding energy -6.3 kcal/mol, (**E**) quercetin-IL-6 with binding energy -7.4 kcal/mol, (**F**) quercetin-TNF with binding energy -6.7 kcal/mol, (**G**) quercetin-MAPK1 with binding energy -8.5 kcal/mol, (**H**) quercetin-1l1B with binding energy -7.9 kcal/mol, (**I**) kaempferol-TNF with binding energy -8.6 kcal/mol, (**J**) naringenin-MAPK1 with binding energy -8.3 kcal/mol.

We mainly simulated the docking of 3 active compounds (quercetin, naringenin, and kaempferol) of bitter almond-licorice with ACE2 (PDB ID: 1R42) (Figs. **8A**-**D**), and the results showed that compared with hydroxychloroquine, these active ingredients combined with ACE2 generally ideal, indicating that these 3 active ingredients had the potential to treat COVID-19. To further verify the therapeutic potential of these 3 active ingredients, we selected 4 targets: IL-6 (PDB ID: 1IL6), TNF (PDB ID: 1A8M), MAPK1 (PDB ID: 1PME), and IL1B (PDB ID: 1I1B), from ten core targets to simulate molecular dockings (Figs. **8E-J**). More structural docking details are shown in Figs. (**9A-F**). Quercetin binds to 1IL6 (IL6) through 2 hydrogen bonds between MET-68 and SER-170. Other forces, including van der Waals forces, pi-sigma bonds, and pi-alkyl were also found. And form 5 hydrogen bonds with ASN-34, ASN-92, ALA-33, AGR-32 and PHE-144 of 1A8M (TNF). Besides, van der Waals, pi-sigma, pi-alkyl, and carbon hydrogen bond also existed. When encountered 1PME (MAPK1), it formed 3 hydrogen bonds with GLN-132, ASN-158 and HIS125, and found pi- sulfur. When the target is 1I1B (1I1B), hydrogen bond, van der Waals force, carbon-hydrogen bond and other forces were also found. The docking results revealed that the 3 key ingredients were successfully docked to the corresponding targets.

## 4 DISCUSSION

COVID-19 is highly contagious and has overwhelmingly surpassed severe acute respiratory syndrome coronavirus (SARS- CoV) and Middle East respiratory syndrome coronavirus (MERS- CoV) in terms of the number of infected individuals and the spatial scope of the affected area, which has posed an extraordinary threat [25]. The control of COVID-19 in China not only benefits from the restriction of personnel mobility but also the extensive application of traditional Chinese medicine has made an indispensable contribution to the prevention and treatment of COVID-19. Chinese medicines such as ShuangHuangLian oral liquids (commercial Chinese medicine) and LianHuaQingWen capsule were used to treat COVID-19, which proved that TCM can effectively alleviate symptoms, improve the cure rate, reduce the death rate, and promote organ recovery in infected individuals [13, 26]. The effective cure rate of Qingfei Paidu Decoction containing bitter almond-licorice for COVID-19 is more than 90% [27], which could relieve symptoms, promote the resolution of lung inflammation, and tend to reduce the degree of multi-organ damage [28]. However, the composition of TCM is complex, and it is often used in combination with other drugs, which leads to the uncertainty of its active ingredients and mechanism in disease treatment. To provide the scientific basis for the treatment of COVID-19 with Chinese medicine, this study illustrated the mechanisms of how the bitter almond-licorice treat COVID-19 by the network pharmacology, and revealed that the 89 active ingredients of the bitter almond-licorice acted on COVID-19 through 102 targets which contained core targets, such as IL6, MAPK1, TNF, and IL1B, and that these active components are involved in numerous biological pathways and molecular interactions in the body, and act synergistically.

The selection of the “herb-ingredient-target” visual network diagram identified the main 3 active ingredients, quercetin, kaempferol, and naringenin, which may play a critical role in the treatment of COVID-19. Quercetin, with the highest degree value has certain preventive or therapeutic effects on various viruses, such as dengue virus infection, murine coronavirus, and human immunodeficiency virus type 1 [29, 30]. Moreover quercetin plays an anti-inflammatory role by inhibiting the production of inflammatory factors [31] such as IL-6, IP-10, TNF-α. Quercetin also increases the oxidative stress-fighting ability of the cells by stimulating the synthesis and expression of antioxidant enzymes, such as catalase, glutathione peroxidase, and superoxide dismutase, which protects the tissues from oxidative damage and injury as expression is enhanced [32]. In addition, oral intake of quercetin in humans is well tolerated with a very low incidence of adverse effects [33]. Quercetin may be a potential medicine for the treatment of COVID-19. Kaempferol, a flavonoid, has a protective effect on dysfunctional cells by regulating endoplasmic reticulum stress and autophagy [34]. In addition, kaempferol has anti-inflammatory, anti-oxidative stress, and anti-viral effects, which could significantly decrease the release of histamine, IL-6, IL-8, IL-1β and TNF-α in activated HMC-1 mast cells. Besides, it can inhibit the activation of IKKβ, inhibit the phosphorylation of IκBα, and prevent NF-κB from entering the nucleus, thus affecting the release of related inflammatory mediators [35, 36]. Unluckily, kaempferol is poorly absorbed, with an extremely poor oral bioavailability [37]. But the combination of kaempferol and quercetin can enhance the therapeutic effect of quercetin by blocking the efflux of quercetin [38]. Naringenin can act on macrophages to activate the anti-inflammatory response factor, Nrf2 [39]. This suggests that bitter almond-licorice may act on COVID-19 through antiviral mechanisms, and regulation of inflammatory response.

The PPI network revealed that IL-6, MAPK1, TNF, and IL1B were the main targets of bitter almond-licorice to interfere with COVID-19. Clinical studies have shown abnormal concentrations of different relevant cytokines in patients with COVID-19 [40], such as IL-6, TNF-α, IL-1β. IL-6 is a cytokine that contributes to host defense against various infections and tissue damage [41]. However, the excessive amount of IL-6 may cause a cytokine storm during the anti-infection process [42]. Cytokine storm is an abnormal immune activation caused by viruses which may interfere with the body's immune system, leading to diffuse acute lung injury, impairment of ventilation function of lung and a series of critical manifestations [42-44]. IL-6 is considered to be one of the potential biomarkers of COVID-19 progression, and is a very important guideline for patient progression, treatment, and prognosis assessment [45]. TNF-α is a classic pro-inflammatory cytokine whose prolonged elevated or excessive production can trigger cytokine storm leading to cell death during the acute phase of tissue injury [46]. IL-1β is also an intense pro-inflammatory cytokine which may cause cell damage [47]. MAPK1 is an important member of the MAPKs family. MAPK is a serine/threonine kinase which is widely expressed in the central nervous system, where it plays a key role in extracellular signal transduction and cellular responses by regulating important processes such as cell proliferation, differentiation, growth and apoptosis in response to stimulation by various extracellular factors [48].

The results of GO enrichment analysis showed that bitter almond-licorice mainly regulates cell stress, cytokine activity and receptor ligand binding. KEGG signaling pathway analysis indicated that bitter almond-licorice was involved in the IL-17 signaling pathway, TNF signaling pathway and Th17 cell differentiation in the treatment of COVID-19, all of which were closely related to the inflammation process. The molecular docking results showed that the main effective components of bitter almond-licorice, quercetin, kaempferol, and naringenin, had a good ability with the corresponding target protein.

This study revealed the mechanism of bitter almond-licorice in the treatment of COVID-19 through network pharmacology and molecular docking, and provided a certain theoretical basis for the clinical application of bitter almond-licorice.

However, there are several limitations to this study. First, network pharmacology and molecular docking depend on existing datasets which are influenced by databases. Moreover, network pharmacology cannot predict the process of medicine in the body, and more experiments are required to validate the predicted conclusion.

## CONCLUSION

The network pharmacology and molecular docking were used to reveal that bitter almond-licorice treats COVID-19 through multi-ingredients, multi-targets, and multi-pathways and the mechanism of bitter almond-licorice treatment of COVID-19 may serve a potential therapeutic purpose by inhibiting inflammatory responses and regulating cellular stress. However, this work is a prospective study based on data mining, and the findings need to be interpreted with caution.

## Figures and Tables

**Fig. (1):**
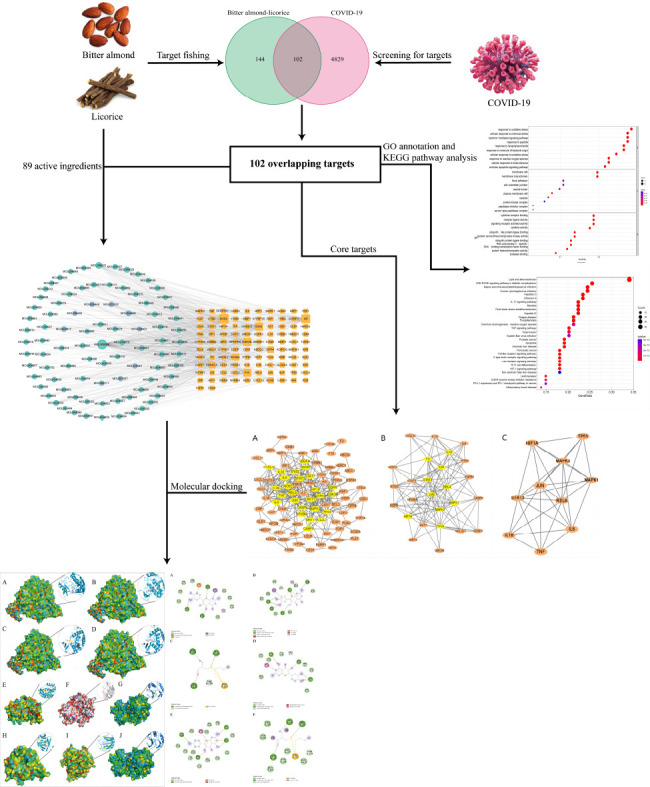
Workflow of the study design.

**Fig. (9):**
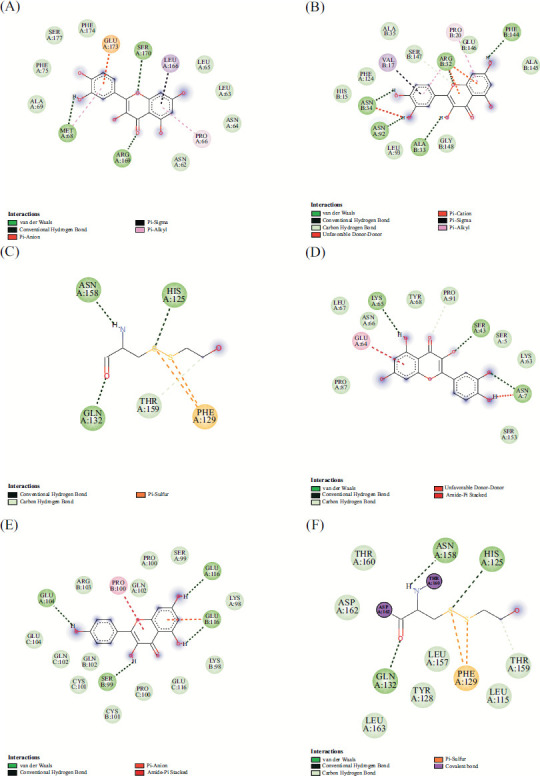
The active site ligation of three compounds of bitter almond-licorice with related targets. (**A**) quercetin-IL-6, (**B**) quercetin-TNF, (**C**) quercetin-MAPK1, (**D**) quercetin-1l1B, (**E**) kaempferol-TNF, (**F**) naringenin-MAPK1.

**Table 1:** The information on potential targets of bitter almond-licorice against COVID-19.

**S. No.**	**UniProt ID**	**Gene Symbol**	**S. No.**	**UniProt ID**	**Gene Symbol**	**S. No.**	**UniProt ID**	**Gene Symbol**
1	Q14524	SCN5A	35	P06213	INSR	69	P11021	HSPA5
2	P17612	PRKACA	36	P30048	PRDX3	70	P04626	ERBB2
3	P31645	SLC6A4	37	P09488	GSTM1	71	Q13085	ACACA
4	P35228	NOS2	38	P03973	SLPI	72	Q03135	CAV1
5	P49841	GSK3B	39	P27361	MAPK3	73	P13726	F3
6	P10275	AR	40	P28482	MAPK1	74	P17302	GJA1
7	P27487	DPP4	41	P49327	FASN	75	P01584	IL1B
8	P24941	CDK2	42	P01130	LDLR	76	P13500	CCL2
9	P18031	PTPN1	43	P04040	CAT	77	P10145	CXCL8
10	P15121	AKR1B1	44	P04114	APOB	78	P29474	NOS3
11	Q04206	RELA	45	P04035	HMGCR	79	P01137	TGFB1
12	P47989	XDH	46	P36956	SREBF1	80	P60568	IL2
13	P78380	OLR1	47	P33527	ABCC1	81	P00750	PLAT
14	P00742	F10	48	Q15848	ADIPOQ	82	P07204	THBD
15	P22303	ACHE	49	P17174	GOT1	83	P05121	SERPINE1
16	P05412	JUN	50	P23141	CES1	84	P01579	IFNG
17	P05112	IL4	51	P35610	SOAT1	85	P01583	IL1A
18	Q96EB6	SIRT1	52	P40763	STAT3	86	P05164	MPO
19	P06576	ATP5F1B	53	P11802	CDK4	87	P11388	TOP2A
20	Q96Q05	IKBKB	54	P08254	MMP3	88	Q9UNQ0	ABCG2
21	P31749	AKT1	55	P00533	EGFR	89	Q16236	NFE2L2
22	P10415	BCL2	56	P15692	VEGFA	90	P09874	PARP1
23	Q07812	BAX	57	Q07817	BCL2L1	91	O14625	CXCL11
24	P01375	TNF	58	Q03405	PLAU	92	P19875	CXCL2
25	O95433	AHSA1	59	P22301	IL10	93	P02741	CRP
26	P42574	CASP3	60	P05231	IL6	94	P02778	CXCL10
27	P03956	MMP1	61	P04637	TP53	95	O15111	CHUK
28	P42224	STAT1	62	P25963	NFKBIA	96	P10451	SPP1
29	P06493	CDK1	63	P16435	POR	97	P07339	CTSD
30	P09601	HMOX1	64	Q14790	CASP8	98	P17936	IGFBP3
31	P08684	CYP3A4	65	P11387	TOP1	99	P29965	CD40LG
32	P05362	ICAM1	66	P04049	RAF1	100	P10914	IRF1
33	Q16678	CYP1B1	67	Q16665	HIF1A	101	P21860	ERBB3
34	P35869	AHR	68	Q9UII4	HERC5	102	P55786	NPEPPS

**Table 2:** The target of quercetin, naringenin and kaempferol.

**Mol ID**	**Molecule Name**	**Degree ** **Centrality**	**Target**
MOL000098	Quercetin	78	SCN5A, PRKACA, NOS2, AR, DPP4, AKR1B1, RELA, XDH, F10, ACHE, JUN, AKT1, BCL2, BAX, TNF, AHSA1, CASP3, MMP1, STAT1, CDK1, HMOX1, CYP3A4, ICAM1, CYP1B1, AHR, INSR, PRDX3, GSTM1, MAPK1, MMP3, EGFR, VEGFA,
MOL000098	Quercetin	78	BCL2L1, PLAU, IL10, IL6, TP53, NFKBIA, POR, CASP8, TOP1, RAF1, HIF1A, HERC5, HSPA5, ERBB2, ACACA, CAV1, F3, GJA1, IL1B, CCL2, CXCL8, NOS3, TGFB1, IL2, PLAT, THBD, SERPINE1, IFNG, IL1A, MPO, TOP2A, ABCG2, NFE2L2, PARP1, CXCL11, CXCL2, CRP, CXCL10, CHUK, SPP1, CTSD, IGFBP3, CD40LG, IRF1, ERBB3, PEPPS
MOL000422	Kaempferol	28	PRKACA, NOS2, NOS2, AR, DPP4, RELA, XDH, ACHE, JUN, IKBKB, AKT1, BCL2, BAX, TNF, AHSA1, CASP3, MMP1, STAT1, CDK1, HMOX1, CYP3A4, ICAM1, CYP1B1, AHR, INSR, PRDX3, GSTM1, SLPI
MOL004328	Naringenin	18	PRKACA, RELA, AKT1, BCL2, CASP3, MAPK3, MAPK1, FASN, LDLR, CAT, APOB, HMGCR, SREBF1, ABCC1, ADIPOQ, GOT1, CES1, SOAT1

## Data Availability

All data analyzed in this study are included in the article or its supplementary files.

## LIST OF ABBREVIATIONS

ACE2: Angiotensin-converting Enzyme 2
BP: Biological Process
CC: Cellular Component
COVID-19: Corona Virus Disease 2019
DL: Drug-likeness
GO: Gene Ontology
MERS-CoV: Middle East Respiratory Syndrome Coronavirus
MF: Molecular Function
PPI: Protein-protein Interaction
SARS-CoV: Severe Acute Respiratory Syndrome Coronavirus
TCM: Traditional Chinese Medicine
