# Supplementary material for: Potential Targets and Mechanisms of Bitter Almond-Licorice for COVID-19 Treatment Based on Network Pharmacology and Molecular Docking
Source: Curr Pharm Des. 2023 Dec 13;29(33):2655–67. doi: 10.2174/0113816128265009231102063840 (PMC10788922; doi:10.2174/0113816128265009231102063840)
Supplement: Supplementary file 1 — Supplementary material is available on the publisher’s website along with the published article. [file CPD-29-2655_SD1.pdf]

Supplementary Material

Potential Targets and Mechanisms of Bitter Almond-Licorice for COVID-19 Treatment Based on Network Pharmacology and Molecular Docking

Qiwei Hong[1,2,#], Xinyue Shang[1,2,#], Yanan Wu[1,2], Zhenlin Nie[3], and Bangshun He[1,3,*]

[1]Department of Laboratory Medicine, Nanjing First Hospital, China Pharmaceutical University, Nanjing, China; [2]Department of Laboratory Medicine, Nanjing First Hospital, Nanjing Medical University, Nanjing, China; [3]Department of Clinical Pharmacy, School of Basic Medicine and Clinical Pharmacy, China Pharmaceutical University, Nanjing, China;

1873-4286/23 $65.00+.00 © 2023 Bentham Science Publishers

104 Active Indigents of the Licorice-Bitter Almond Drug Pair

Source	Mol ID	Molecule Name	OB(%)	DL
Bitter almond	MOL012922	l-SPD	87.35	0.54
Bitter almond	MOL010922	Diisooctyl succinate	31.62	0.23
Bitter almond	MOL010921	estrone	53.56	0.32
Bitter almond	MOL007207	Machiline	79.64	0.24
Bitter almond	MOL005030	gondoic acid	30.7	0.2
Licorice	MOL005020	dehydroglyasperins C	53.82	0.37
Licorice	MOL005018	Xambioona	54.85	0.87
Licorice and Bitter almond	MOL005017	Phaseol	78.77	0.58
Licorice	MOL005016	Odoratin	49.95	0.3
Licorice	MOL005013	18α-hydroxyglycyrrhetic acid	41.16	0.71
Licorice	MOL005012	Licoagroisoflavone	57.28	0.49
Licorice	MOL005008	Glycyrrhiza flavonol A	41.28	0.6
Licorice	MOL005007	Glyasperins M	72.67	0.59
Licorice	MOL005003	Licoagrocarpin	58.81	0.58
Licorice	MOL005001	Gancaonin H	50.1	0.78
Licorice	MOL005000	Gancaonin G	60.44	0.39
Licorice	MOL004996	gadelaidic acid	30.7	0.2
Licorice	MOL004993	8-prenylated eriodictyol	53.79	0.4
Licorice	MOL004991	7-Acetoxy-2-methylisoflavone	38.92	0.26
Licorice	MOL004990	7,2',4'-trihydroxy-5-methoxy-3-arylcoumarin	83.71	0.27
Licorice	MOL004989	6-prenylated eriodictyol	39.22	0.41
Licorice	MOL004988	Kanzonol F	32.47	0.89
Licorice	MOL004985	icos-5-enoic acid	30.7	0.2
Source	Mol ID	Molecule Name	OB(%)	DL
Licorice	MOL004980	Inflacoumarin A	39.71	0.33
Source	Mol ID	Molecule Name	OB(%)	DL
Licorice	MOL004978	2-[(3R)-8,8-dimethyl-3,4-dihydro-2H-pyrano[6,5-f]chromen-3-yl]-5-methoxyphenol	36.21	0.52
Licorice	MOL004974	3'-Methoxyglabridin	46.16	0.57
Licorice	MOL004966	3'-Hydroxy-4'-O-Methylglabridin	43.71	0.57
Licorice	MOL004961	Quercetin der.	46.45	0.33
Licorice	MOL004959	1-Methoxyphaseollidin	69.98	0.64
Licorice	MOL004957	HMO	38.37	0.21
Licorice	MOL004949	Isolicoflavonol	45.17	0.42
Licorice	MOL004948	Isoglycyrol	44.7	0.84
Licorice	MOL004945	(2S)-7-hydroxy-2-(4-hydroxyphenyl)-8-(3-methylbut-2-enyl)chroman-4-one	36.57	0.32
Licorice	MOL004941	(2R)-7-hydroxy-2-(4-hydroxyphenyl)chroman-4-one	71.12	0.18
Licorice	MOL004935	Sigmoidin-B	34.88	0.41

**Source**	**Mol ID**	**Molecule Name**	**OB(%)**	**DL**
Licorice	MOL004924	(-)-Medicocarpin	40.99	0.95
Licorice	MOL004917	glycyroside	37.25	0.79
Licorice	MOL004915	Eurycarpin A	43.28	0.37
Source	Mol ID	Molecule Name	OB(%)	DL
Licorice	MOL004913	1,3-dihydroxy-9-methoxy-6-benzofurano[3,2-c]chromenone	48.14	0.43
Licorice	MOL004912	Glabrone	52.51	0.5
Source	Mol ID	Molecule Name	OB(%)	DL
Licorice	MOL004911	Glabrene	46.27	0.44
Licorice	MOL004910	Glabranin	52.9	0.31
Licorice and Bitter almond	MOL004908	Glabridin	53.25	0.47
Licorice	MOL004907	Glyzaglabrin	61.07	0.35
Licorice	MOL004905	3,22-Dihydroxy-11-oxo-delta(12)-oleanene-27-alpha-methoxycarbonyl-29-oic acid	34.32	0.55
Licorice	MOL004904	licopyranocoumarin	80.36	0.65
Licorice and Bitter almond	MOL004903	liquiritin	65.69	0.74
Licorice	MOL004898	(E)-3-[3,4-dihydroxy-5-(3-methylbut-2-enyl)phenyl]-1-(2,4-dihydroxyphenyl)prop-2-en-1-one	46.27	0.31
Licorice	MOL004891	shinpterocarpin	80.3	0.73
Licorice	MOL004885	licoisoflavanone	52.47	0.54
Licorice	MOL004884	Licoisoflavone B	38.93	0.55
Licorice	MOL004883	Licoisoflavone	41.61	0.42
Licorice	MOL004882	Licocoumarone	33.21	0.36
Licorice	MOL004879	Glycyrin	52.61	0.47

Source	Mol ID	Molecule Name	OB(%)	DL
Licorice	MOL004866	2-(3,4-dihydroxyphenyl)-5,7-dihydroxy-6-(3-methylbut-2-enyl)chromone	44.15	0.41
Licorice	MOL004864	5,7-dihydroxy-3-(4-methoxyphenyl)-8-(3-methylbut-2-enyl)chromone	30.49	0.41
Licorice	MOL004863	3-(3,4-dihydroxyphenyl)-5,7-dihydroxy-8-(3-methylbut-2-enyl)chromone	66.37	0.41
Licorice	MOL004860	licorice glycoside E	32.89	0.27
Licorice	MOL004857	Gancaonin B	48.79	0.45
Licorice	MOL004856	Gancaonin A	51.08	0.4
Licorice	MOL004855	Licoricone	63.58	0.47
Licorice	MOL004849	3-(2,4-dihydroxyphenyl)-8-(1,1-dimethylprop-2-enyl)-7-hydroxy-5-methoxy-coumarin	59.62	0.43
Licorice	MOL004848	licochalcone G	49.25	0.32
Licorice and Bitter almond	MOL004841	Licochalcone B	76.76	0.19
Licorice	MOL004838	8-(6-hydroxy-2-benzofuranyl)-2,2-dimethyl-5-chromenol	58.44	0.38
Licorice	MOL004835	Glypallichalcone	61.6	0.19
Licorice	MOL004833	Phaseolinisoflavan	32.01	0.45
Licorice	MOL004829	Glepidotin B	64.46	0.34
Licorice	MOL004828	Glepidotin A	44.72	0.35
Licorice	MOL004827	Semilicoisoflavone B	48.78	0.55

Source	Mol ID	Molecule Name	OB(%)	DL
Licorice	MOL004824	(2S)-6-(2,4-dihydroxyphenyl)-2-(2-hydroxypropan-2-yl)-4-methoxy-2,3-dihydrofuro[3,2-g]chromen-7-one	60.25	0.63
Licorice	MOL004820	kanzonols W	50.48	0.52
Licorice	MOL004815	(E)-1-(2,4-dihydroxyphenyl)-3-(2,2-dimethylchromen-6-yl)prop-2-en-1-one	39.62	0.35
Licorice	MOL004814	Isotrifoliol	31.94	0.42
Licorice	MOL004811	Glyasperin C	45.56	0.4
Licorice	MOL004810	glyasperin F	75.84	0.54
Licorice	MOL004808	glyasperin B	65.22	0.44
Licorice	MOL004806	euchrenone	30.29	0.57
Licorice	MOL004805	(2S)-2-[4-hydroxy-3-(3-methylbut-2-enyl)phenyl]-8,8-dimethyl-2,3-dihydropyrano[2,3-f]chromen-4-one	31.79	0.72
Bitter almond	MOL004355	Spinasterol	42.98	0.76
Licorice	MOL004328	naringenin	59.29	0.21
Licorice	MOL003896	7-Methoxy-2-methyl isoflavone	42.56	0.2
Licorice	MOL003656	Lupiwighteone	51.64	0.37
Bitter almond	MOL003410	Ziziphin_qt	66.95	0.62
Licorice	MOL002565	Medicarpin	49.22	0.34

Source	Mol ID	Molecule Name	OB(%)	DL
Bitter almond	MOL002372	(6Z,10E,14E,18E)-2,6,10,15,19,23-hexamethyltetracosa-2,6,10,14,18,22-hexaene	33.55	0.42
Licorice and Bitter almond	MOL002311	Glycyrol	90.78	0.67
Bitter almond	MOL002211	11,14-eicosadienoic acid	39.99	0.2
Licorice	MOL001792	DFV	32.76	0.18
Licorice	MOL001484	Inermine	75.18	0.54
Bitter almond	MOL000953	CLR	37.87	0.68
Licorice	MOL000500	Vestitol	74.66	0.21
Licorice	MOL000497	Licochalconea	40.79	0.29
Bitter almond	MOL000492	(+)-catechin	54.83	0.24
Bitter almond	MOL000449	Stigmasterol	43.83	0.76
Licorice	MOL000422	kaempferol	41.88	0.24
Licorice	MOL000417	Calycosin	47.75	0.24
Licorice	MOL000392	formononetin	69.67	0.21
Licorice and Bitter almond	MOL000359	sitosterol	36.91	0.75
Licorice	MOL000354	isorhamnetin	49.6	0.31
Licorice	MOL000239	Jaranol	50.83	0.29
Licorice and Bitter almond	MOL000211	Mairin	55.38	0.78
Licorice	MOL000098	quercetin	46.43	0.28
